# Septin Assembly and Remodeling at the Cell Division Site During the Cell Cycle

**DOI:** 10.3389/fcell.2021.793920

**Published:** 2021-11-25

**Authors:** Joseph Marquardt, Xi Chen, Erfei Bi

**Affiliations:** Department of Cell and Developmental Biology, Perelman School of Medicine, University of Pennsylvania, Philadelphia, PA, United States

**Keywords:** septins, septin-associated proteins, RhoGEF, anillin, Elm1, Bud3, Bud4

## Abstract

The septin family of proteins can assemble into filaments that further organize into different higher order structures to perform a variety of different functions in different cell types and organisms. In the budding yeast *Saccharomyces cerevisiae*, the septins localize to the presumptive bud site as a cortical ring prior to bud emergence, expand into an hourglass at the bud neck (cell division site) during bud growth, and finally “split” into a double ring sandwiching the cell division machinery during cytokinesis. While much work has been done to understand the functions and molecular makeups of these structures, the mechanisms underlying the transitions from one structure to another have largely remained elusive. Recent studies involving advanced imaging and *in vitro* reconstitution have begun to reveal the vast complexity involved in the regulation of these structural transitions, which defines the focus of discussion in this mini-review.

## Introduction

Septins are GTP-binding proteins that assemble into heteropolymers that can organize into various filament-containing structures such as rings, hourglasses, and gauzes in different cell types (Byers and Goetsch, 1976a; Kim et al., 1991; Frazier et al., 1998; Lippincott et al., 2001; Ihara et al., 2005; Kissel et al., 2005; Rodal et al., 2005; John et al., 2007; Sirajuddin et al., 2007; Tada et al., 2007; Xie et al., 2007; Bertin et al., 2008; Ong et al., 2014; Renshaw et al., 2014; Karasmanis et al., 2019; Wang et al., 2019). As such, they are considered the fourth cytoskeletal component along with microfilaments, intermediate filaments, and microtubules (Mostowy and Cossart, 2012). Septins play critical roles in cytokinesis, exocytosis, mitosis, ciliogenesis, and cell morphogenesis by acting as a cellular scaffold and/or diffusion barrier (Longtine et al., 1996; Gladfelter et al., 2001; Weirich et al., 2008; McMurray and Thorner, 2009; Oh and Bi, 2011; Mostowy and Cossart, 2012; Dolat et al., 2014). Not surprisingly, mutations in human septin genes have been linked to several diseases including male infertility, cancer, and neurodegenerative diseases (Hall and Russell, 2004; Roeseler et al., 2009; Lin et al., 2011; Dolat et al., 2014; Ageta-Ishihara and Kinoshita, 2021).

Septins are conserved in eukaryotes except higher land plants (Pan et al., 2007). In humans, there are 13 septin genes whose products can form several different combinations of heterooligomers (usually octamers) depending on the tissue type in which they are expressed (Kinoshita, 2003; Pan et al., 2007; Estey et al., 2010; Sellin et al., 2011; Mendonca et al., 2019; Soroor et al., 2021). Additionally, many septin genes, especially *SEPT9*, code for multiple isoform variants (Robertson et al., 2004; Hall et al., 2005; Estey et al., 2010; Connolly et al., 2011a; Connolly et al., 2011b; Sellin et al., 2012). Such a complexity in septin expression and assembly has hampered the rapid progress in the analysis of human septins. Model organisms have had a major impact on our understanding of septin biology with much of the emphasis placed on the budding yeast *Saccharomyces cerevisiae*. There are a total of seven septin genes in *S. cerevisiae*, five of which (*CDC3, CDC10, CDC11, CDC12,* and *SHS1*) are expressed in mitotically active cells (Hartwell, 1971; Byers and Goetsch, 1976b; Carroll et al., 1998; Mino et al., 1998) and the other two (*SPR3* and *SPR28*) are expressed during meiosis (Ozsarac et al., 1995; De Virgilio et al., 1996; Fares et al., 1996). The limited number of septin genes, coupled with the ease of genetic manipulation and easily scored phenotypes associated with septin malfunction, make budding yeast an excellent model organism for studying the regulation of septin organization.

To understand how septin high order structures are regulated, the precise organization of their building blocks must be known. In *S. cerevisiae*, the mitotic septins oligomerize into hetero-octamers comprised of a core Cdc12-Cdc3-Cdc10-Cdc10-Cdc3-Cdc12 hexamer with either Cdc11 or Shs1 at the terminal ends (Bertin et al., 2008; Garcia et al., 2011; Khan et al., 2018). The presence of Cdc11 or Shs1 can influence what higher order structures form *in vitro*, with Cdc11-capped octamers more likely to form paired filaments from end-on-end Cdc11 interactions (Frazier et al., 1998; Bertin et al., 2008) and Shs1-capped octamers to laterally associate into curved bundles and rings (Garcia et al., 2011). With Cdc11 and Shs1 both expressed in mitotic cells, it is possible that the regulated combination of differentially capped octamers could produce the distinct structures *in vivo*, from the nascent ring at the presumptive bud site, to the hourglass at the bud neck, and finally the double ring surrounding the cytokinesis machinery during cell division (Bertin et al., 2012; Ong et al., 2014; Mcquilken et al., 2017; Weems and McMurray, 2017; Marquardt et al., 2019).

The septin architecture is dynamically remodeled at the division site during the cell cycle, and this involves the regulation by post-translational modifications (PTMs) and septin-associated proteins (SAPs) (Gladfelter et al., 2001; McMurray and Thorner, 2009; Hernández-Rodríguez and Momany, 2012; Alonso et al., 2015; Perez et al., 2016). Many yeast septins have PTMs such as phosphorylation, acetylation, and sumoylation added or removed at precise times, which may change their architectural organization [for full reviews on these modifications see (Hernández-Rodríguez and Momany, 2012; Perez et al., 2016; Marquardt et al., 2019)]. In fact, several protein kinases such as the LKB1-like Elm1 and the MARK/PAR1-related Gin4 not only depend on septins for their localization to the bud neck, but also influence the stability or functionality of the septin structures present there (Longtine et al., 1998; Bouquin et al., 2000; Mortensen et al., 2002; Asano et al., 2006). Besides the PTMs, different SAPs are also involved in the regulation of septin organization during the cell cycle. For example, Bni5 is associated with the septin hourglass before cytokinesis (Lee et al., 2002; Fang et al., 2010) and can bundle septin filaments *in vitro* (Patasi et al., 2015; Booth et al., 2016). The Rho guanine nucleotide-exchange factor (RhoGEF) Bud3 and the anillin-like protein Bud4 can stabilize the double ring structure during cytokinesis (Wloka et al., 2011; Eluere et al., 2012; Mcquilken et al., 2017).

In this review, we will summarize and draw conclusions from recent work that has begun to illustrate the regulation that occurs at the transition times between both the nascent ring to hourglass (Marquardt et al., 2020) and the hourglass to double ring structures (Chen et al., 2020). These different structures each have specific functions at their respective stages during the cell cycle and the precise transformations over a relatively short time ensure that these functions are ordered appropriately. While we are beginning to elucidate the pathways involved in the regulation of these structural transitions, much work remains to fully comprehend the mechanisms of septin assembly and remodeling in yeast and beyond.

## Transforming the Nascent Septin Ring Into a Septin Hourglass

Upon starting a new cell cycle, haploid cells develop a new bud site axial to the previous division site (Chant and Pringle, 1995; Pringle et al., 1995). Through a series of feedback loops involving the Rho-like small GTPase Cdc42, its GEFs, and GTPase-activating proteins, septin recruitment, and targeted exocytosis, a nascent ring is formed at the new budding site, and the growth of the bud begins (Gladfelter et al., 2002; Caviston et al., 2003; Iwase et al., 2006; Okada et al., 2013). The septin ring then expands into an hourglass spanning the bud-neck region. This septin hourglass serves as both a scaffold for the assembly of cytokinesis machinery such as the actomyosin ring (AMR) (Bi et al., 1998; Lippincott and Li, 1998; Fang et al., 2010; Schneider et al., 2013; Finnigan et al., 2015) and numerous other protein complexes such as the morphogenesis checkpoint cascade (Barral et al., 1999; Shulewitz et al., 1999; Longtine et al., 2000; Cid et al., 2001b) and as a membrane diffusion barrier to compartmentalize the mother and bud plasma membranes (Barral et al., 2000; Luedeke et al., 2005; Shcheprova et al., 2008). As a cellular scaffold, it is not surprising that the septin hourglass is highly stable when analyzed by fluorescence recovery after photo bleaching (FRAP), which is in stark contrast to the relatively mobile nature of the nascent ring during its initial assembly process (Caviston et al., 2003; Dobbelaere et al., 2003). It remains unclear whether an altered self-assembly state of septins and/or the addition of new SAPs during hourglass expansion accounts for this increased stability.

### Understanding Paired and Unpaired Septin Filament Assembly and Organization *in vitro*

**FIGURE 1:**
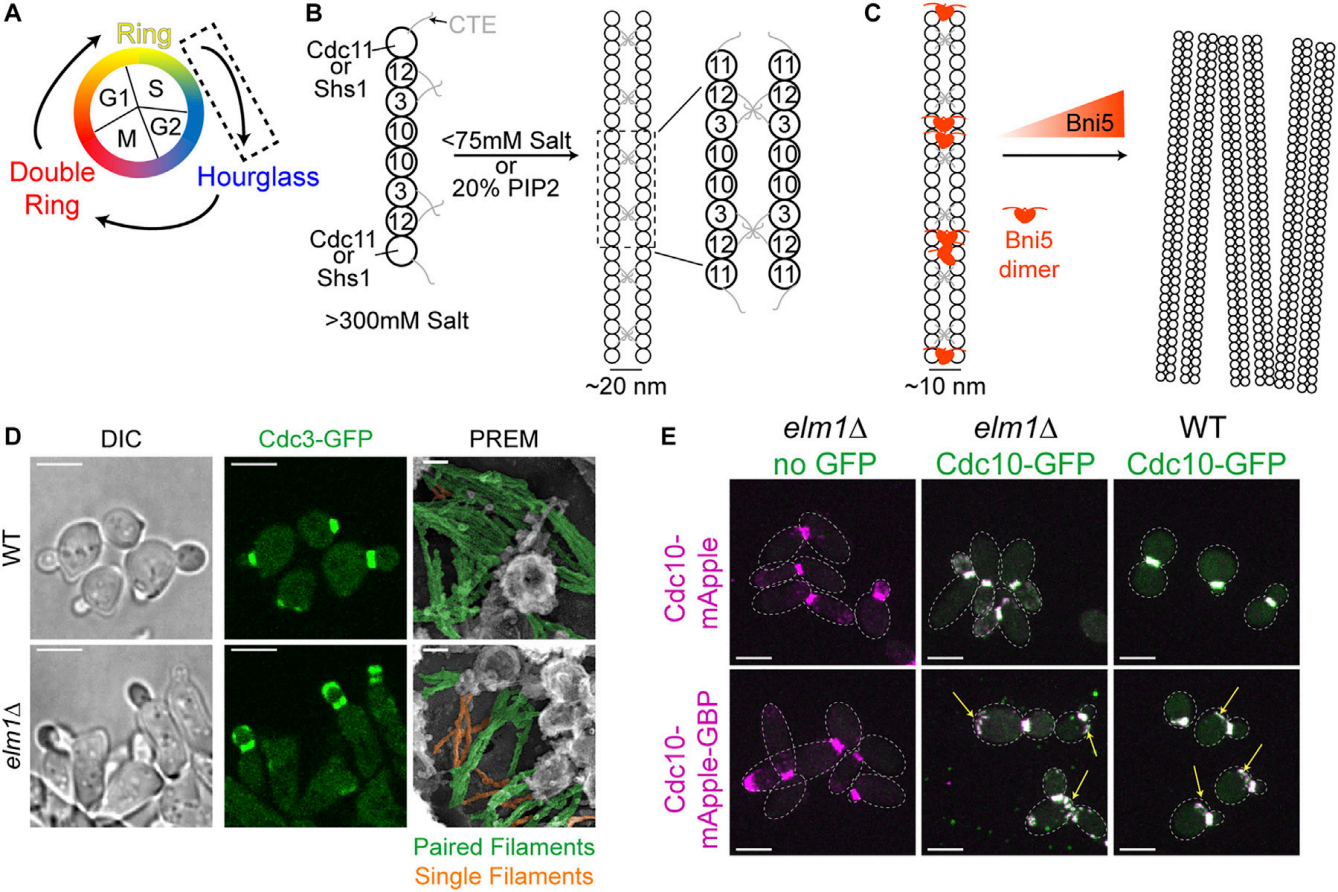
Transitioning from a dynamic ring to a stable hourglass. **(A)** Depiction of different septin structures visualized during the yeast cell cycle. Arrows indicate transitions occurring between two structures. Dashed box indicates that the focus of this figure is on the nascent ring-to-hourglass transition that occurs during bud emergence and expansion. **(B)** **Left**, depiction of septin rod assembly in high salt (>300 mM KCl or NaCl) *in vitro* (Frazier et al., 1998; Bertin et al., 2008). **Right**, depiction of Cdc11-capped rods forming paired filaments in either low salt (<75 mM) (Bertin et al., 2008) or on the surface of PIP2-containing lipid monolayers (Bertin et al., 2010; Bridges et al., 2014); dashed box indicates zoomed in inset shown to the right. **(C)** **Left**, depiction of two Bni5 proteins (red molecules) interacting with Cdc11-CTEs found in paired septin filaments to lower the average interfilament distance to 10 nm (Finnigan et al., 2015; Booth et al., 2016). **Right**, depiction of Bni5 bundling septin filaments into large clusters *in vitro* in a dose-dependent manner (Patasi et al., 2015). **(D)** Representative images of WT **(top)** or *elm1Δ* **(bottom)** cells synchronized in G1 by α-factor arrest and released for 1.5 h to obtain small-to medium-sized buds. **Left** and **middle panels** show DIC and fluorescence imaging of Cdc3 (green), respectively; scale bars = 5 µm. **Right panels** show PREM images of samples with paired filaments pseudo-colored green and single filaments pseudo-colored orange; scale bars = 50 nm. Images taken from (Marquardt et al., 2020). **(E)** Representative images of indicated strains with Cdc10-mApple **(top row)** or Cdc10-mApple-GBP **(bottom row)** in magenta and Cdc10-GFP, which is expressed exogenously from a *CEN* plasmid, in green. Images are maximum projections. Yellow arrow indicates septin ring from previous cell cycle. Dotted line is cell periphery. Scale bars = 5 µm. Images taken from (Marquardt et al., 2020).

To understand how a stable hourglass can form from a relatively dynamic septin ring (Figure 1A), one must first understand how septins are assembled into filaments, which are further organized into higher-order structures such as rings and hourglasses. Yeast septins were first seen at the bud neck as a membrane-associated structure made from filaments with a 10 nm width (Byers and Goetsch, 1976a). However, when expressed and purified *in vitro* from *E. coli* or budding yeast in the presence of high salt (>300 mM KCl or NaCl) only septin rods of the approximate length of an octamer were formed (Figure 1B, left) (Frazier et al., 1998; Bertin et al., 2008). When the ionic strength was lowered (50–75 mM salt) and with Cdc11 serving as the terminal septin subunit were filaments visualized (Frazier et al., 1998; Bertin et al., 2008), with the C-terminal extensions (CTEs) of Cdc3 and Cdc12 interacting on neighboring filaments to form the paired filaments (Figure 1B, right) (Bertin et al., 2008; Bertin et al., 2010). In contrast, the Shs1-capped septin rods associate laterally into curved bundles and rings *in vitro* under low salt condition (Garcia et al., 2011). While data from this study concerning the ability for Shs1-capped septin rods to assemble end-on-end into filaments is not as clear as that of Cdc11-capped septin rods, additional analysis by Förster Resonance Energy Transfer has more conclusively negated the possibility that two Shs1-capped rods can polymerize end-on-end (Booth et al., 2015). Given that the budding yeast cytoplasm has potassium and sodium concentrations of 200–300 and 20 mM, respectively (van Eunen et al., 2010; Cyert and Philpott, 2013), the ability of yeast septins to spontaneously generate filaments in the cytoplasm should be quite low.

Strikingly, in the presence of lipid monolayers containing 20% phosphatidylinositol-4,5-bisphosphate (PIP2), purified Cdc11-capped septin rods could form long paired septin filaments even at higher salt concentrations (Figure 1B, right) (Bertin et al., 2010; Bridges et al., 2014). This implies that septin octamers probably exist in the cytoplasm and are assembled into paired filaments once bound to the plasma membrane at the presumptive bud site or the bud neck. This indeed appears to be the case, as it was found by fluorescence correlation spectroscopy that septins in the cytoplasm exist as complexes and not as monomers or filaments (Bridges et al., 2014). The septin complexes appear to “sense” and favor the membrane curvature found at the bud neck, as the purified septin complexes preferentially engaged with lipid-coated beads of diameters producing a similar positive curvature as that found at the bud neck (Bridges et al., 2016; Cannon et al., 2019). The organization of the septin filaments into higher-order structures may also be governed by the changes in membrane curvature during bud growth and cytokinesis, as suggested by a recent study that examined and modeled the impact of varying degrees of positive and negative curvature on septin filament assembly and organization on lipid-covered surfaces *in vitro* (Beber et al., 2019).

Higher-order assemblies of septins are likely regulated by PTMs as well as SAPs (Gladfelter et al., 2001; Mcmurray and Thorner, 2009; Hernández-Rodríguez and Momany, 2012; Alonso et al., 2015; Perez et al., 2016). One such SAP, Bni5, localizes to the bud neck in a septin-dependent manner (Lee et al., 2002). Bni5 appears to interact with the CTEs of the terminal subunits Cdc11 and Shs1 *in vitro* and *in vivo* (Figure 1C) (Finnigan et al., 2015; Booth et al., 2016). Recombinant Bni5 can reduce the distance between individual filaments of a paired filament and can also bundle septin filaments, thereby contributing to a higher-order organization (Figures 1B,C) (Patasi et al., 2015; Booth et al., 2016). It is possible that a combination of phospholipid composition, PTMs, and SAPs is able to bypass the need for positive curvature during the nascent septin ring formation, as there is no discernable bud neck at this point and the membrane topology at the presumptive bud site does not match the curvature preference of the septins. Taken together, the *in vitro* analysis of the ability of septins to interact with membranes, recognize membrane curvature, and recruit SAPs for higher-order organization will undoubtedly help understand the mechanisms for the assembly and regulation of the plethora of septin structures *in vivo*.

### A LKB1-Like Kinase Acts as a Regulator of Septin Filament Pairing to Control Hourglass Assembly and Stabilization

Upon bud emergence or shortly after, the septins transition from a dynamic nascent ring at the presumptive bud site to a stable septin hourglass at the bud neck (Caviston et al., 2003; Dobbelaere et al., 2003). This stabilization could be caused by filament pairing as observed by thin-section EM and EM tomography (Byers and Goetsch, 1976a; Bertin et al., 2012; Bertin and Nogales, 2012) and by polarized fluorescence microscopy (Vrabioiu and Mitchison, 2006; Demay et al., 2011). These studies are not in agreement as to the orientation of the paired filaments in relation to the mother-bud axis, but are all in agreement that the hourglass seems to be made up of mainly paired filaments. This was further confirmed by our group in cell cortices visualized by platinum-replica electron microscopy (PREM) (Ong et al., 2014). The PREM data favors the orientation of radial paired filaments (about 300–400 nm in length) in an “early hourglass” being parallel to the mother-bud axis as postulated by the groups using polarized fluorescence microscopy (Vrabioiu and Mitchison, 2006; Demay et al., 2011), because the presumed same paired filaments in a “late or transitional hourglass” lie perpendicular to the visualized AMR that runs circumferentially at the bud neck (Ong et al., 2014). Despite the consensus on the paired filaments making up the septin hourglass, whether and how filament pairing is regulated *in vivo* has remained elusive.

We recently provided the first evidence of such regulation using genetic perturbations and PREM analyses of the septin hourglass *in vivo* (Marquardt et al., 2020). The protein kinase Elm1 localizes to the bud neck from bud emergence to the onset of cytokinesis in a septin dependent manner (Bouquin et al., 2000; Kang et al., 2016; Marquardt et al., 2020). This localization pattern matches perfectly with the timing of septin hourglass assembly and maintenance at the bud neck. Elm1 had already been implicated in maintaining the stability of septin structures at the bud neck (Bouquin et al., 2000; Asano et al., 2006), but the underlying mechanism had remained unknown at the time. We discovered that the septin hourglass was selectively destabilized with mostly the daughter half of the hourglass mislocalized to the growing bud cortex in *elm1Δ* mutants in both a kinase-dependent and -independent manner (Figure 1D, left and center panels) and that these phenotypes are at least partially mediated through Bni5 (Marquardt et al., 2020). This destabilization appears to be largely due to an increase in single septin filaments comprising the septin hourglass-like structure when visualized by PREM (Figure 1D, right panels) (Marquardt et al., 2020). This was confirmed by artificially pairing septin filaments using the GFP-nanobody/binding protein (GBP) system (Rothbauer et al., 2006; Kubala et al., 2010) and witnessing a significant rescue of the septin phenotype at the hourglass stage in *elm1Δ* cells (Figure 1E) (Marquardt et al., 2020). The same experiment also provided evidence that septin filament pairing and unpairing are likely regulated throughout the cell cycle, because aberrant septin structures at the old division site remained even in otherwise wild-type (WT) cells when the septins were forced to stay paired (Figure 1E, arrows) (Marquardt et al., 2020). Thus, to understand the structural transitions or architectural remodeling, one must understand the mechanisms of septin filament pairing and unpairing during the cell cycle.

## Transforming the Septin Hourglass Into a Double Ring

**FIGURE 2:**
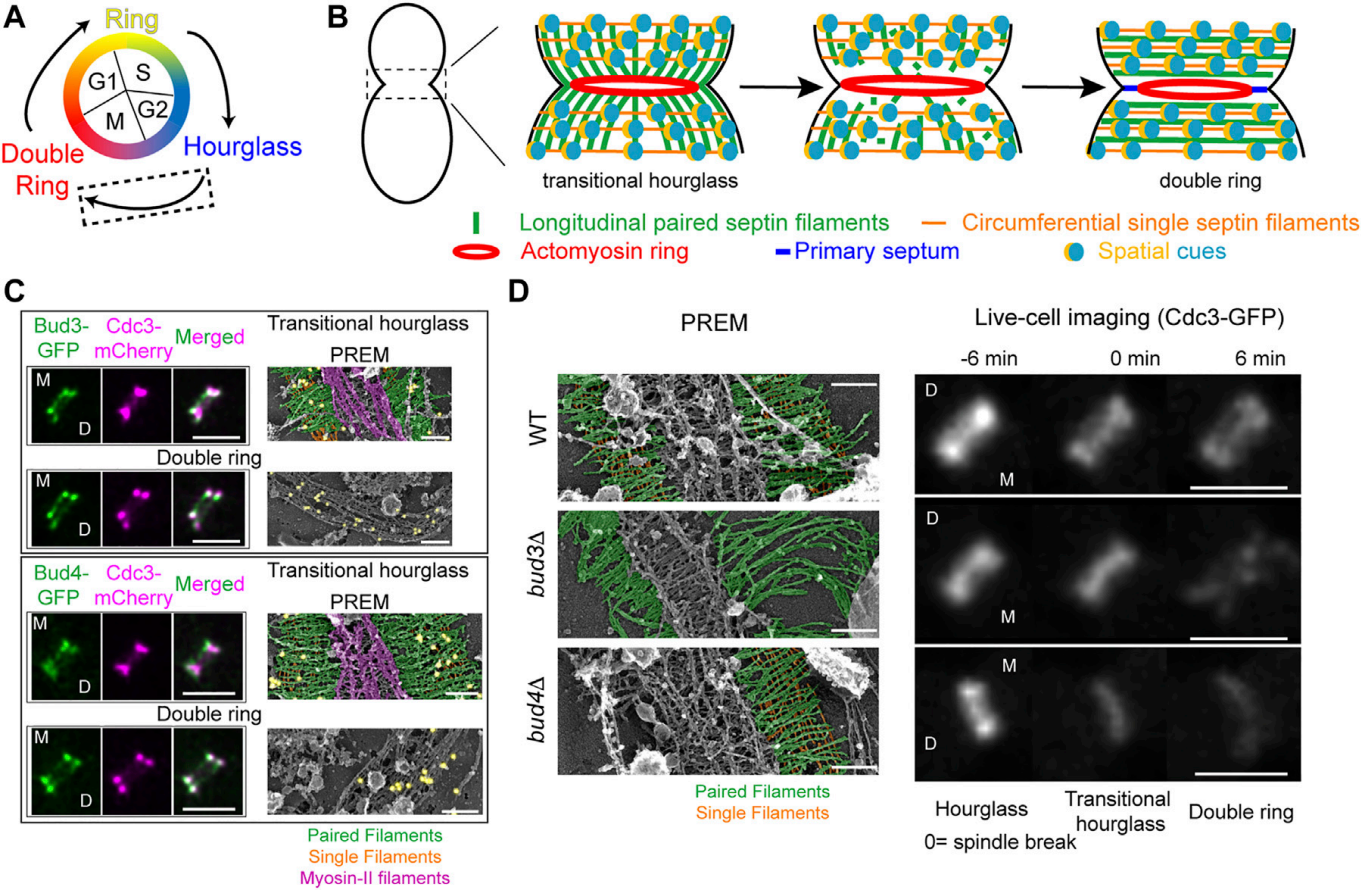
Transitioning from an hourglass to a double ring. **(A)** Depiction of different septin structures visualized during the yeast cell cycle. Arrows indicate transitions occurring between two structures. Dashed box indicates that the focus of this figure is on the hourglass-to-double ring transition that occurs at the onset of cytokinesis. **(B)** A model of septin architectural remodeling at the division site. **(C)** Bud3 **(green, top)** and Bud4 **(green, bottom)** localization in relation to the septin hourglass and double ring (magenta) at the bud neck by iSIM. Scale bars = 2 µm. Immunogold labeling PREM analysis of Bud3 **(yellow, top)** and Bud4 **(yellow, bottom)** localization in relation to the septin transitional hourglass and double ring **(right panels)**. Scale bar = 200 nm. Paired filaments (green), single filaments (orange), and myosin-II filaments (purple). Images taken from (Chen et al., 2020). **(D)** PREM analysis of the transitional hourglass **(left panels)** with paired filaments pseudo-colored green and single filaments pseudo-colored orange; scale bars = 200 nm. Images taken from (Chen et al., 2020). Representative images of septin organization (Cdc3-GFP) at the bud neck during the HDR transition in WT **(top)**, *bud3Δ* **(middle)**, and *bud4Δ* **(bottom)** cells **(right panels)**. Scale bars = 2 µm. Images taken from (Chen et al., 2020).

At the onset of cytokinesis in *S. cerevisiae*, the septin hourglass undergoes a dramatic architectural remodeling by “splitting” into two separate rings (Figure 2A), which allows the AMR to access the plasma membrane and initiate its constriction (Kim et al., 1991; Cid et al., 2001a; Lippincott et al., 2001; Tamborrini et al., 2018). A similar transition occurs in the fission yeast *Schizosaccharomyces pombe* (Berlin et al., 2003; Tasto et al., 2003) as well as the filamentous fungus *Aspergillus nidulans* (Westfall and Momany, 2002). In mammalian cells (Renshaw et al., 2014; Karasmanis et al., 2019; Wang et al., 2019), septins also undergo reorganization from an hourglass-like structure during cleavage furrow ingression to a double ring during abscission. Despite the striking similarity of septin architectural remodeling at the division site across species, the molecular mechanisms underlying this process remain largely unknown in any system.

### Spatially Controlled Septin Filament Disassembly and Reorganization During the Hourglass-to-Double Ring Transition

Two major events are associated with the septin hourglass-to-double ring (HDR) transition: septin filament disassembly and reorganization. FRAP analysis indicates that septins become more dynamic during the HDR transition (Caviston et al., 2003; Dobbelaere et al., 2003). This dynamic change is accompanied by the net loss of septins at the division site by 20–30%, implicating septin filament disassembly in the process (Caviston et al., 2003; Dobbelaere et al., 2003; Demay et al., 2011; Wloka et al., 2011). Analysis by polarized fluorescence microscopy suggests that paired septin filaments reorganize from a radial arrangement in the hourglass to a circumferential arrangement in the double ring, a 90° change in filament orientation (Vrabioiu and Mitchison, 2006; Demay et al., 2011). Despite these insights, the detailed architectures of the septin hourglass and double ring at the filament level and the molecular mechanisms controlling the HDR transition remained unknown.

By combining cell synchronization with PREM, we have determined the architecture of different septin assemblies at the division site during the cell cycle (Ong et al., 2014; Chen et al., 2020; Marquardt et al., 2020). The “early hourglass” from pre-anaphase cells contains exclusively paired filaments arranged radially and parallel to the mother-bud axis (Ong et al., 2014). This structure is converted into a “transitional hourglass” before cytokinesis, in which the paired filaments are connected perpendicularly by periodic circumferential single septin filaments on the membrane-proximal side (Ong et al., 2014). Strikingly, these intersecting septin filaments, which define a septin gauze, are located exclusively at the outer zones of the transitional hourglass whereas myosin-II filaments occupy the middle zone but exclusively on the cytoplasmic side of the hourglass (Figure 2B, left) (Ong et al., 2014; Chen et al., 2020). At the onset of cytokinesis and under the control of the mitotic exit network (Cid et al., 2001a; Lippincott et al., 2001; Ong et al., 2014; Tamborrini et al., 2018; Chen et al., 2020), this “zonal” transitional hourglass is remodeled into a double ring that consists of exclusively circumferential paired and single septin filaments (Figure 2B, right) (Ong et al., 2014; Chen et al., 2020). These distinct architectures revealed by PREM analysis provide a foundational blueprint for mechanistic analysis of septin high-order assembly and remodeling.

In addition to septin filament disassembly (Figure 2B, center) (Caviston et al., 2003; Dobbelaere et al., 2003; Demay et al., 2011; Wloka et al., 2011), careful analysis of septin behavior during the HDR transition by FRAP, photo-activation, photoconversion, and super-resolution microscopy suggests that there must be “spatial cues” present at the ends of the septin hourglass that controls septin filament reassembly and reorganization into a double ring (Figure 2B) (Ong et al., 2014). What are the spatial cues? How do they control the HDR transition? These questions remained unanswered.

### A RhoGEF and an Anillin Act Together as the Spatial Cues to Drive the Septin Hourglass-to-Double Ring Transition

Our recent work suggests that the RhoGEF Bud3 and the anillin-like protein Bud4 function as the spatial cues to drive the HDR transition (Chen et al., 2020). Bud3 and Bud4 were initially identified as factors essential for axial budding in *S. cerevisiae* (Chant et al., 1995; Sanders and Herskowitz, 1996). Bud3 also acts as a GEF for Cdc42 in early G1 phase to spatially link successive polarization or budding events in haploid cells (Kang et al., 2014). Bud3 and Bud4 interact with each other (Kang et al., 2012; Kang et al., 2013; Wu et al., 2015) and associate with the septin hourglass after S/G2 and with the septin double ring after the HDR transition in a nearly identical pattern (Figure 2C, left) (Chant et al., 1995; Sanders and Herskowitz, 1996; Wloka et al., 2011; Kang et al., 2013; Chen et al., 2020). PREM coupled with immunogold labeling indicates that Bud3 and Bud4 specifically associate with the septin gauze at the outer zones of the transitional hourglass (Figure 2C, right) (Chen et al., 2020). Both proteins have been implicated in septin organization, likely at the stage of double ring formation (Lord et al., 2000; Gladfelter et al., 2005; Guo et al., 2011; Wloka et al., 2011; Eluere et al., 2012; Kang et al., 2013; Wu et al., 2015; Mcquilken et al., 2017). Taken together, these observations suggest that Bud3 and Bud4 likely define the spatial cues that act in the right place at the right time to drive the HDR transition, but how?

Live-cell imaging and PREM analyses demonstrate that Bud3 and Bud4 play distinct and essential roles in controlling the septin HDR transition (Chen et al., 2020). As indicated above, the transitional hourglass in WT cells possesses a zonal architecture where the myosin-II filaments are sandwiched by Bud3 and Bud4-decorated septin gauze (Figure 2C; Figure 2D, top row, left). During the HDR transition, the septins in the middle of the hourglass are preferentially lost whereas those at the ends of the hourglass are preferentially stabilized (Figure 2D, top row, right), leading to the double ring formation. In the absence of Bud3, however, the circumferential single filaments at the ends of the transitional hourglass are completely lost, with many of the structures displaying elongated paired filaments at one side of the hourglass (Figure 2D, middle row, left) (Chen et al., 2020). This filament-level observation may explain the phenotype of *bud3Δ* cells detected by live-cell imaging that, during the HDR transition, the septin hourglass was first thinned at its edges, followed by the disintegration or fragmentation of the remaining structure in the middle (Figure 2D, middle row, right) (Chen et al., 2020). These observations suggest that Bud3 is specifically required for the stabilization of the transitional hourglass at its edges, and, furthermore, this selective stabilization mechanism must act before the cell cycle-triggered disassembly of the septin filaments in the middle of the hourglass (located between the PM and the myosin-II filaments). In the absence of Bud4, the entire transitional hourglass became destabilized, often in an asymmetric manner where one side of the structure was affected more than the other (Figure 2D, bottom row, left) (Chen et al., 2020). This is consistent with live-cell imaging analysis showing that the mother side of the septin double ring is preferentially lost during the HDR transition (Wloka et al., 2011; Chen et al., 2020). In the absence of both Bud3 and Bud4, the transitional hourglass was severely compromised and, consequently, was hardly detectable by PREM and the double ring was essentially abolished (Chen et al., 2020). Taken together, these data suggest that Bud3 and Bud4 function as the spatial cues by exclusively localizing to the outer zones of the transitional hourglass and instructing the double ring formation at the ends of the hourglass. Bud3 is essential for the circumferential single filament assembly whereas Bud4 is likely required for the stability of both paired and single filaments in the transitional hourglass, especially at the mother side of the bud neck.

## Discussion

How a cell assembles a septin structure at a discrete membrane site and how the structure is remodeled *in situ* into a distinct architecture to perform specific functions are central questions in the septin field that remain largely unanswered. Based on the collective data presented above, it is safe to say that the transitions from the nascent ring to the hourglass and the hourglass to double ring require precise regulation by specific SAPs. Recent works have placed Elm1 as a regulator of septin filament pairing to stabilize the early septin hourglass at the onset of bud formation (Marquardt et al., 2020) and Bud3 and Bud4 as the spatial cues at the ends of a late hourglass to reorganize and stabilize both single and paired filaments during the HDR transition (Chen et al., 2020). By utilizing genetic perturbations and following septin kinetics via confocal microscopy and septin filament architecture *via* PREM, these studies have begun to elucidate the mechanisms of septin organization *in vivo* at an unprecedented resolution.

While Elm1 is known to regulate septin filament pairing during hourglass formation (Marquardt et al., 2020), the underlying mechanism remains unknown. Could Elm1 directly phosphorylate the septins or another SAP to induce the change from single to paired filaments, or does Elm1 oppose an unknown molecule that destabilizes septin filament pairing? As septins tend to form paired filaments *in vitro* either in solution under low-salt condition or on lipid monolayer even under high-salt condition (Frazier et al., 1998; Bertin et al., 2008; Bertin et al., 2010; Bridges et al., 2014), we favor the latter possibility of an indirect regulation by antagonizing a destabilization factor. However, other studies have shown that Elm1 can phosphorylate SAPs with known roles in septin stability including Bni5 (Patasi et al., 2015), Gin4 (Asano et al., 2006), and the morphogenetic checkpoint kinase Hsl1 (Szkotnicki et al., 2008), leaving the possibility open for non-mutually exclusive pathways of action.

Protein kinases have been found to regulate septin structural stability in other systems including at the annulus of spermatozoa and at the base of dendritic spines (Brand et al., 2012; Shen et al., 2017; Yadav et al., 2017; Lin et al., 2019). Similar to the LKB1-like kinase Elm1, LKB1 regulates glucose metabolism by phosphorylating AMP-activated protein kinase (AMPK) (Hawley et al., 2003; Hong et al., 2003; Woods et al., 2003; Shaw et al., 2004; Rubenstein et al., 2006; Mccartney et al., 2016), and has a conserved function in polarity (Watts et al., 2000; Nakano and Takashima, 2012). However, there is no evidence thus far that LKB1 or other Elm1 “homologues” regulate septin architecture in other systems.

Bud3 and Bud4 are known to localize exclusively to the gauze-like structure at the outer zones of the transitional hourglass (Chen et al., 2020). However, it remains a mystery how this unique localization is achieved and how it facilitates the HDR transition. Does the Bud3 and Bud4 complex function as the membrane anchor to stabilize the single filaments at the ends of the transitional hourglass during the paired filament disassembly, and, at the same time, also function as the spatial landmark to recruit septin complexes and/or short filaments from the disassembled paired filaments and reassemble and reorganize them into a double ring? How Bud3 and Bud4 interact with each other and with the septins also remains unknown.

The HDR transition is triggered by the activation of the mitotic exit network (Cid et al., 2001a; Lippincott et al., 2001; Tamborrini et al., 2018) and is accompanied by 90° change in the orientation of the paired filaments (Vrabioiu and Mitchison, 2006; Demay et al., 2011; Ong et al., 2014). Whether and how Bud3 and Bud4 are involved in this process remains unknown. All these questions must be addressed in order to achieve a mechanistic understanding of the HDR transition.

Like the Bud3-Bud4 module in budding yeast, a RhoGEF-anillin module appears to be involved in the coordination of septin remodeling and cytokinesis in many organisms including fission yeast (Berlin et al., 2003; Tasto et al., 2003; Munoz et al., 2014; Wang et al., 2015), *Drosophila* (Hickson and O’Farrell, 2008), and mammalian cells (Frenette et al., 2012; Renshaw et al., 2014; Karasmanis et al., 2019; Wang et al., 2019). Whether this module represents a conserved core mechanism for septin remodeling during cytokinesis across model systems requires further investigation.
